# Supplementary material for: Greater than X kb: a quantitative assessment of preservation conditions on genomic DNA quality, and a proposed standard for genome-quality DNA
Source: PeerJ. 2016 Oct 11;4:e2528. doi: 10.7717/peerj.2528 (PMC5068448; doi:10.7717/peerj.2528)
Supplement: Supplemental Information 1 [file peerj-04-2528-s001.pdf]

Supplementary Information: Mulcahy et al., PeerJ

Protocol for using ImageJ to determine relative band size of DNA from gel images

1. **Download Program**: Download Java program ImageJ from http://rsb.info.nih.gov/ij
2. **Open Image**: Open raw image file in ImageJ with ⌘o or using the menu (File>Open).
3. **Invert Image**: Invert the image with shift+i. This is not necessary, but I find that it is easier to see the limits of bands with an inverted image, and it makes the density curves that we will create positive rather than negative. It also makes Step 4 (image leveling) significantly easier.
4. **Level Image**: Make sure lanes in your image are horizontally level using the menu (Image>Transform>Rotate). This brings up a dialog that allows you to rotate the image by degrees (positive numbers for clockwise rotation, negative numbers for counterclockwise rotation). To determine if rotation is necessary, select the Preview box, set the Angle to 0, and increase the Grid Lines value. This allows you to compare your lanes to the horizontal and vertical lines of the grid. Change the Angle value until the lanes in your image are horizontally level (i.e. you want the top bands in both ladders to be level).
5. **Subtract Background**: Subtract background from image using the menu (Process>Subtract Background). A pop-up window will open. Make sure that the box for Light Background is checked, as well as the box for Preview. Choose the Rolling ball radius that gives the whitest background without degrading DNA signal. Usually the default (50.0 pixels) works fine, but you may want to play with this. If you use too low of a value, you start to lose dark pixels at the edges of the DNA bands, but you want the lowest value obtainable without losing dark pixels.
6. **Select Rectangle Tool**: Select the Rectangle tool from the toolbar
7. **Draw Box Encompassing Ladder**: Draw a rectangle on the lane of the first ladder. The bands of the ladder should cross the entire width of the drawn box, so make the box as narrow as possible. Vertically, the box should encompass the entire DNA band of the lane on your gel with the greatest vertical range (but do not include the well in the box). In other words, there should be no white space on the left or right sides between the DNA band and the sides of the drawn box, but there should be white space between the longest band and the top of the box, and white space between the shortest band and the bottom of the box. If you make a mistake in drawing boxes or selecting lanes and need to start over, this can be done via the menu (Analyze>Gels>Reset)
8. **Select Box as First Lane**: Select this lane as the first lane to be analyzed using ⌘1. This can also be accomplished using the menu (Analyze>Gels>Select First Lane)
9. **Select Next Lane**: Drag the box to the next lane to be measured, and select this lane as the next to be analyzed using ⌘2. This can also be accomplished using the menu (Analyze>Gels>Select Second Lane). When you drag the box to the

new lane, carefully select the location of the new lane horizontally, attempting to center the box on the bands (as in Step 8). You do not have to be as careful with vertical placement of the box: ImageJ will automatically align new boxes vertically as you select them.

10. **Select all Lanes:** Repeat Step 9 for all lanes that you wish to analyze. Make sure that the last lane selected is the last ladder. It doesn't matter which ladder is selected first, and which is selected last, as long as they are the first and last selected.
11. **Create Intensity Plot**: Create intensity plot of the selected lanes using ⌘3. This can also be accomplished using the menu (Analyze>Gels>Plot). If you need to remake the intensity plot, close the current plot, make the gel image the active window, and use the menu (Analyze>Gels>Re-Plot Lanes). This will give you a new, clean intensity plot.
12. **Draw Line for DNA Size Cutoff:** From the toolbar, choose the Straight tool. Draw vertical line on the intensity plot from the apex of the ladder peak of choice on the first ladder (top of page) to the apex of the ladder peak of choice on the second ladder (bottom of page). This separates the intensity curves of each lane into a region greater than the size of the peak chosen and a region less than the size of the peak chosen.
13. **Close All Regions to Measure**: All regions under the intensity curves must be closed to measure area, but you will notice that the right side of the intensity curve does not meet the vertical line at the right side of the plot, leaving this region open. To fix this, with the Vertical tool still selected, draw a vertical line from the furthest right part of the curve on the first ladder to the furthest right part of the curve on the last ladder. This may have to be repeated on the left side of the curve if it doesn't meet the left side of the plot. You will know if this is necessary if, during Step 14, the Wand tool highlights more than just the area under the curve.
14. **Select Region Above Cut-off for First Measurement**: Choose the Wand tool from the toolbar. Select the region under the intensity curve on the left side of the vertical line from Step 12 for your first sample.
15. **Measure First Region**: Some users may find that an area measurement is automatically taken when the region is selected using the Wand tool. If a new box (labeled "Results") appears when you select a region, then measurements are automatic. If no "Results" box appears, you need to tell ImageJ to measure each region with ⌘m, or using the menu (Analyze>Measure). The number in the results window is the area of the curve greater than the size of the peak chosen. If you need to measure multiple areas (i.e. the curve reaches the lower limit of the graph, essentially splitting up the region), select each while holding shift, then measure after all regions have been selected. If measurements are automatic, you cannot select multiple regions before measuring. Instead, measure each region separately and add them together to get the area of the entire curve
16. **Select and Measure Region Below Cut-Off**: Repeat Steps 14 and 15 with the region under the intensity curve on the right side of the vertical line. This is the area of the curve less than the size of the peak chosen, and should appear in the "Results" window, below the first value. Note that ImageJ numbers the

measurements sequentially as they are made, so keep track of which value belongs to each curve area measurement.

17. **Measure All Regions**: Repeat Step 14 through Step 16 for each sample.
18. **Save Results**: Save the Results table with ⌘s or using the menu (File>Save As). By default, ImageJ saves the table as a tab-delimited file with an excel (.xls) extension.
19. **Calculate Proportion:** For each sample, add the two portions (left and right) to get the total area under the curve. Divide the first area value (area to the left of the peak) by the total area to obtain the % of area under the curve greater than the size chosen.

Notes:
This protocol is for usage on a Mac, usage on a PC is similar (replace ⌘ with Ctrl for keyboard shortcuts).

The ImageJ users guide can be viewed at http://rsb.info.nih.gov/ij/docs/guide/index.html or downloaded using http://rsb.info.nih.gov/ij/docs/guide/user-guide.pdf.
This protocol was based on the video tutorial found at http://imagejdocu.tudor.lu/doku.php?id=video:analysis:gel_quantification_analysis
All video tutorials can be accessed at http://imagejdocu.tudor.lu/doku.php?id=video:start
